# Regulation of Neutrophil NADPH Oxidase, NOX2: A Crucial Effector in Neutrophil Phenotype and Function

**DOI:** 10.3389/fcell.2022.945749

**Published:** 2022-07-14

**Authors:** Marie-Hélène Paclet, Salomé Laurans, Sophie Dupré-Crochet

**Affiliations:** ^1^ Univ. Grenoble Alpes, CNRS, UMR 5525, VetAgro Sup, Grenoble INP, CHU Grenoble Alpes, TIMC, T-RAIG, Grenoble, France; ^2^ Université Paris-Saclay, CNRS UMR 8000, Institut de Chimie Physique, Orsay, France

**Keywords:** NADPH oxidase (NOX2), phagocytose, phosphoinositides, metabolism, neutrophil

## Abstract

Reactive oxygen species (ROS), produced by the phagocyte NADPH oxidase, NOX2, are involved in many leukocyte functions. An excessive or inappropriate ROS production can lead to oxidative stress and tissue damage. On the other hand, an absence of ROS production due to a lack of a functional NADPH oxidase is associated with recurrent infections as well as inflammation disorders. Thus, it is clear that the enzyme NADPH oxidase must be tightly regulated. The NOX2 complex bears both membrane and cytosolic subunits. The membrane subunits constitute the flavocytochrome *b*_558_, consisting of gp91^phox^ (Nox2) and p22^phox^ subunits. The cytosolic subunits form a complex in resting cells and are made of three subunits (p47^phox^, p40^phox^, p67^phox^). Upon leukocyte stimulation, the cytosolic subunits and the small GTPase Rac assemble with the flavocytochrome *b*_558_ in order to make a functional complex. Depending on the stimulus, the NADPH oxidase can assemble either at the phagosomal membrane or at the plasma membrane. Many studies have explored NOX2 activation; however, how this activation is sustained and regulated is still not completely clear. Here we review the multiple roles of NOX2 in neutrophil functions, with a focus on description of its components and their assembly mechanisms. We then explain the role of energy metabolism and phosphoinositides in regulating NADPH oxidase activity. In particular, we discuss: 1) the link between metabolic pathways and NOX2 activity regulation through neutrophil activation and the level of released ROS, and 2) the role of membrane phosphoinositides in controlling the duration of NOX2 activity.

## Introduction: NADPH Oxidase Roles in Neutrophil Functions

The first discovered role of ROS produced by NOX2 was pathogen killing. Upon phagocytosis the NADPH oxidase is activated and produces superoxide anions, O_2_^.-^, inside the phagosome as soon as the phagosome has formed (Tlili et al., 2011). Inside the phagosome, O_2_^.-^ dismutates into hydrogen peroxide, H_2_O_2_. In neutrophils, the granules release myeloperoxidase (MPO) which catalyzes the formation of hypochlorous acid (HOCl) from chloride ions and H_2_O_2_. Several millimolars of O_2_^.-^ are produced in the phagosome. Neither the ROS chemistry nor the mechanism of toxicity of this species within the peculiar environment of the phagosome have been well defined (Winterbourn et al., 2016).

Evidence for the involvement of ROS in pathogen killing comes largely from people who lack a functional NADPH oxidase as in the chronic granulomatous disease (CGD). In this genetic disease, people are faced with chronic and persistent infections. Neutrophils isolated from these patients are defective in killing bacteria and fungi (Klebanoff, 2005). Moreover, recent data indicate that CGD neutrophils have an increased cytokine production and secretion of the chemoattractant leukotriene B4 when they are challenged with fungus particles (Song et al., 2020a; Yoo et al., 2021). This increased cytokine and leukotriene B4 production probably contributes to the aberrant inflammation observed in CGD patients. The production of H_2_O_2_, thanks to NADPH oxidase activation, modulates the signaling pathways involved in cytokine production although their specific targets have not been identified (Yoo et al., 2021). Among the potential targets, the activity of NF-κB, which is involved in inflammatory mediator transcription, has been shown to undergo a redox regulation (Trevelin et al., 2020).

NADPH oxidase activation is triggered by phagocytosis but also by soluble stimuli. Long-lasting NADPH oxidase activity has been observed *in vitro* in adherent neutrophils stimulated by cytokines or bacterial chemotactic peptides such as N-formyl-methionyl-leucyl-phenylalanine (fMLP) (Fumagalli et al., 2013; McMillan et al., 2014; Song et al., 2020b). Following stimulus of this type, NOX2 assembles at the plasma membrane and produces ROS in the extracellular medium.

This ROS production then regulates the migration of neutrophils (Trevelin et al., 2020). *Kuiper et al.* showed that upon stimulation by a fMLP gradient, the ROS produced inhibit the activity of the lipid phosphatase PTEN (Phosphatase and TENsin homolog) at the cell front (Kuiper et al., 2011). The inhibition occurs through oxidation of cysteine 124 in the catalytic site (Lee et al., 2002). PTEN inhibition prevents the phosphatidylinositol 3,4,5-trisphosphate (PIP_3_) dephosphorylation at the cell front. This PIP_3_ accumulation is a key event for the activation of signaling molecules involved in neutrophil polarization and chemotaxis (Wang, 2009). Furthermore, ROS induce actin glutathionylation which is necessary for chemotaxis *in vitro* and for the recruitment of neutrophils to the site of infection *in vivo* (Sakai et al., 2012).

NADPH oxidase activity has also been shown to be involved in neutrophil apoptosis (Dupre-Crochet et al., 2013) and, more recently, in the formation of neutrophil extracellular traps (NETs) which consist of decondensed chromatin associated with granule proteins such as MPO and neutrophil elastase. The release of NETs can be fatal to neutrophils since they eventually explode to release these NETs. The involvement of ROS in NETs formation seems to depend on the stimulus (Kenny et al., 2017). NETs avert the dissemination of the pathogen; however, produced in excess or in an inappropriate context, NETs may in fact contribute to the disease process (Sollberger et al., 2018). Recently NETs have been described in COVID infections, where they have been shown to contribute to tissue injury and immunothrombosis (Veras et al., 2020; Ackermann et al., 2021).

Moreover, an excessive NADPH oxidase activation can be detrimental for the surrounding tissues. Excess ROS production by neutrophils has been involved in auto-immune diseases (Glennon-Alty et al., 2018) and chronic inflammatory disease states, such as periodontal disease or chronic obstructive pulmonary disease (COPD) (Jasper et al., 2019; Zeng et al., 2019). In COPD, activated neutrophils are recruited to the lungs of the patients. ROS produced by the NADPH oxidase contribute to the oxidative stress, which leads to increased inflammation, cellular senescence, altered organelle functions especially mitochondria functions and DNA damage (Barnes, 2022). These pathophysiological effects of oxidative stress in COPD has been described in recent reviews (Manevski et al., 2020; Barnes, 2022).

Below, we discuss the NADPH oxidase components and their assembly mechanism, and then review the role of energy metabolism and phosphoinositides in regulating the NADPH oxidase activity.

### NADPH Oxidase Components and Assembly

The phagocyte NADPH oxidase is made of two membrane subunits, gp91^phox^/Nox2 and p22^phox^, three cytosolic subunits, p40^phox^, p47^phox^, p67^phox^, and the small GTPase Rac. The membrane-bound NADPH oxidase subunits are located at the plasma membrane and also in the endocytic compartments in macrophages and neutrophils (Casbon et al., 2009; Joly et al., 2020). In these latter cells however most of the membrane subunits of the NADPH oxidase reside in granules (Borregaard et al., 1983; Lominadze et al., 2005). During phagocytosis, granules and endocytic compartments fuse with the phagosome providing it with membrane subunits as well as lytic enzymes. Nox2 is a 91 kDa glycoprotein responsible for NOX2 catalytic activity. It transfers electrons from cytosolic NADPH to O_2_ producing superoxide anions (Vermot et al., 2021). The p22^phox^ subunit stabilizes Nox2 at the plasma membrane and enables Nox2 heme acquisition (DeLeo et al., 2000). In its C-terminus, p22^phox^ bears a proline-rich region (PRR), which interacts with p47^phox^ SH3 (SRC Homology 3) domains and may also interact with the same domain in p40^phox^ (Tamura et al., 2007). In the resting state, the cytosolic subunits p47^phox^, p67^phox^ and p40^phox^ form a heterotrimeric complex. p67^phox^ binds to the PRR domain of p47^phox^ via its SH3 domain. p40^phox^-p67^phox^ interaction involves the PB1 (Phox and Bem1) domains of both proteins (Vermot et al., 2021).

A 3D model of the complex has recently been proposed based on biophysical studies and structural data: the complex has an elongated shape; the non-structured C-ter of p47^phox^ constitutes a flexible region that may facilitate interaction with the membrane subunits (Ziegler et al., 2019). p47^phox^ and p40^phox^ also contain a PX (PhoX homology) domain that interacts with anionic phospholipids of the membrane (see paragraph 2.3 below) (Ellson et al., 2001; Kanai et al., 2001; Karathanassis et al., 2002). However, in the resting state, p47^phox^, like p40^phox^, is in an auto-inhibitory conformation, preventing the formation of the complex (Ago et al., 1999; Karathanassis et al., 2002; Honbou et al., 2007).

NADPH oxidase can be activated by signalling pathways triggered by soluble stimuli such as fMLP or by pathogen phagocytosis. The phagocytosis is promoted by opsonins *i.e.,* immunoglobulins G and complement molecules (C3b and C3bi) that cover the pathogen following antibody production and activation of the complement. Certain agents can potentiate the activation of NOX2. NOX2 is then in a “primed” state and the NOX2 activity is higher after an activation by a second stimulus such as those mentioned above. Such priming agents include some cytokines, chemo-attractants and Toll-like receptor agonists (El-Benna et al., 2016). EL-Benna et al. describe these priming events in detail in the preceding review.

Upon neutrophil stimulation, p47^phox^ specific serines are phosphorylated by different kinases (MAPK, PKC ...) (Belambri et al., 2018) and these phosphorylations unfold p47^phox^. P47^phox^ can then interact with p22^phox^ and anionic phospholipids at the plasma membrane thus mediating the recruitment of p67^phox^ and p40^phox^. Concomitantly, Rac2, highly expressed in neutrophils, dissociates from a GDP dissociation inhibitor (GDI) and exchanges its GDP with GTP. The GTP-bound Rac2 associates with the membrane via its polybasic domain and prenyl group, and binds to p67^phox^ (Bokoch and Diebold, 2002). Rac2-p67^phox^ interaction favours the binding of p67^phox^ to Nox2 and may participate together with p67^phox^, in regulating the electron flow from NADPH to oxygen, leading to superoxide anion production (Nisimoto et al., 1999; Bokoch and Diebold, 2002).

Different studies, including our own, have observed the dynamics of the cytosolic subunits during phagocytosis using time-lapse confocal video-microscopy (Li et al., 2009; Matute et al., 2009; Tlili et al., 2012; Faure et al., 2013; Song et al., 2017). The following model can be proposed: the complex assembly occurs as soon as the phagocytosis starts, then p47^phox^ and Rac2 leave the phagosome within a few minutes, whereas p40^phox^ and p67^phox^ stay until the end of ROS production (Figure 2A, see paragraph 2.3).

Because of their major roles in neutrophil functions, ROS production and NADPH oxidase activity are highly regulated in space and time. This regulation has been extensively studied and depends on the subunit expression (Nunes et al., 2013), the phosphorylation of NOX2 subunits (Belambri et al., 2018), the trafficking of the subunits to the phagosomal or plasma membrane and ion fluxes (Nunes et al., 2013).

In the following section, we will focus on the role of energy metabolism and phosphoinositides in regulating NADPH oxidase activity.

### Interconnection of Neutrophil Energy Metabolism and NADPH Oxidase Activity

NADPH oxidase activation is dependent on cytoskeleton modifications (Bengtsson et al., 1991), on phosphorylation of cytosolic factors, especially p47^phox^, and membrane cytochrome *b*_558_ (Nox2 and p22^phox^) (El Benna et al., 1997; Bouin et al., 1998; Regier et al., 1999; Raad et al., 2009), and on NADPH availability. All these processes require energy.

In physiological conditions, neutrophil cell metabolism depends essentially on glucose and on the glycolytic pathway for ATP production and energy supply (Borregaard and Herlin, 1982; Anderson et al., 1991; Maianski et al., 2004). Treatment of neutrophils with a glycolysis inhibitor completely abolished phorbol myristate acetate (PMA)-induced NOX2 activity (Chacko et al., 2013).

Furthermore, 6-phosphofructo-2-kinase (PFK2), an enzyme involved in glycolysis regulation, has been identified in the active NOX2 complex isolated from neutrophils stimulated with PMA. Inhibition of PFK2 expression leads to a decrease in NOX2 activity, indicating spatial and functional interactions between enzymes involved in energy metabolism and the phagocyte NOX2 complex (Baillet et al., 2017).

However, the story is not so simple! Neutrophils were considered for decades as a homogeneous cell population with a short half-life and a nearly absence of transcriptional activity (Mantovani et al., 2011). However, recent studies reported heterogeneity of neutrophil phenotypes and revealed the highly-developed plasticity of these cells in response to various physiological and pathological conditions (Silvestre-Roig et al., 2019; Yang et al., 2019). This heterogeneity is especially evident in their functions and their capacity to produce reactive oxygen species via NOX2 activation.

When NOX2 is activated, electrons are transferred from the donor, NADPH, to the acceptor, O_2_ then leading to the release of superoxide anions. This process called “oxidative burst” is extremely fast and requires a large amount of NADPH, the NOX2 cofactor. NADPH availability appears to be a key element in spatial and temporal NOX2 activation. NADPH concentration has been shown to oscillate in a wave-like manner in resting neutrophils. In stimulated neutrophils, the amplitude and/or frequency of NADPH oscillations increase according to the nature of the stimulus (Petty, 2001). Changes in stimulus-induced NADPH oscillations have been correlated with abnormality of NOX2-derived ROS production in neutrophils from patients suffering from chronic inflammatory disorders. This observation suggests a link between NADPH concentration and NOX2 activity (Petty, 2001).

The main source of NADPH in neutrophils is the glucose-dependent pentose phosphate pathway (PPP). Activation of neutrophils with various stimuli leads to an increase in PPP metabolites (Britt et al., 2022). In the PPP oxidative phase, the enzymes glucose-6-phosphate dehydrogenase (G6PD) and 6-phosphogluconate dehydrogenase (6 PGDH) catalyse the two steps leading to NADPH generation (Curi et al., 2020). Activity of G6PD and 6 PGDH is involved in NOX2 activity regulation. Patients with severe G6PD deficiency are more susceptible to infections and present dysfunctions in neutrophil microbicidal mechanisms (Gray et al., 1973). Moreover, G6PD deficiency may result in an absence of ROS production by PMA-stimulated neutrophils (Tsai et al., 1998). At the molecular level, G6PD and 6 PGDH form a supramolecular complex mainly localized at the periphery of neutrophils. This localization facilitates the interaction with the G6PD substrate *i.e.,* glucose-6-phosphate, produced at the plasma membrane, and thus the production of NADPH (Kindzelskii et al., 2004). Interestingly, in neutrophils from pregnant women, the complex G6PD/6 PGDH is relocalized to the microtubule-organizing-center, modifying the site of NADPH release. This difference correlates with the decrease in NOX2-derived ROS production observed in neutrophils from pregnant women (Kindzelskii et al., 2004). Excitingly, proteomic analysis of the constitutively active NOX2 complex isolated from neutrophils has revealed the association of 6 PGDH with the active NOX2 complex and the role of 6 PGDH in the modulation of ROS production via NADPH availability (Baillet et al., 2011).

**FIGURE 1:**
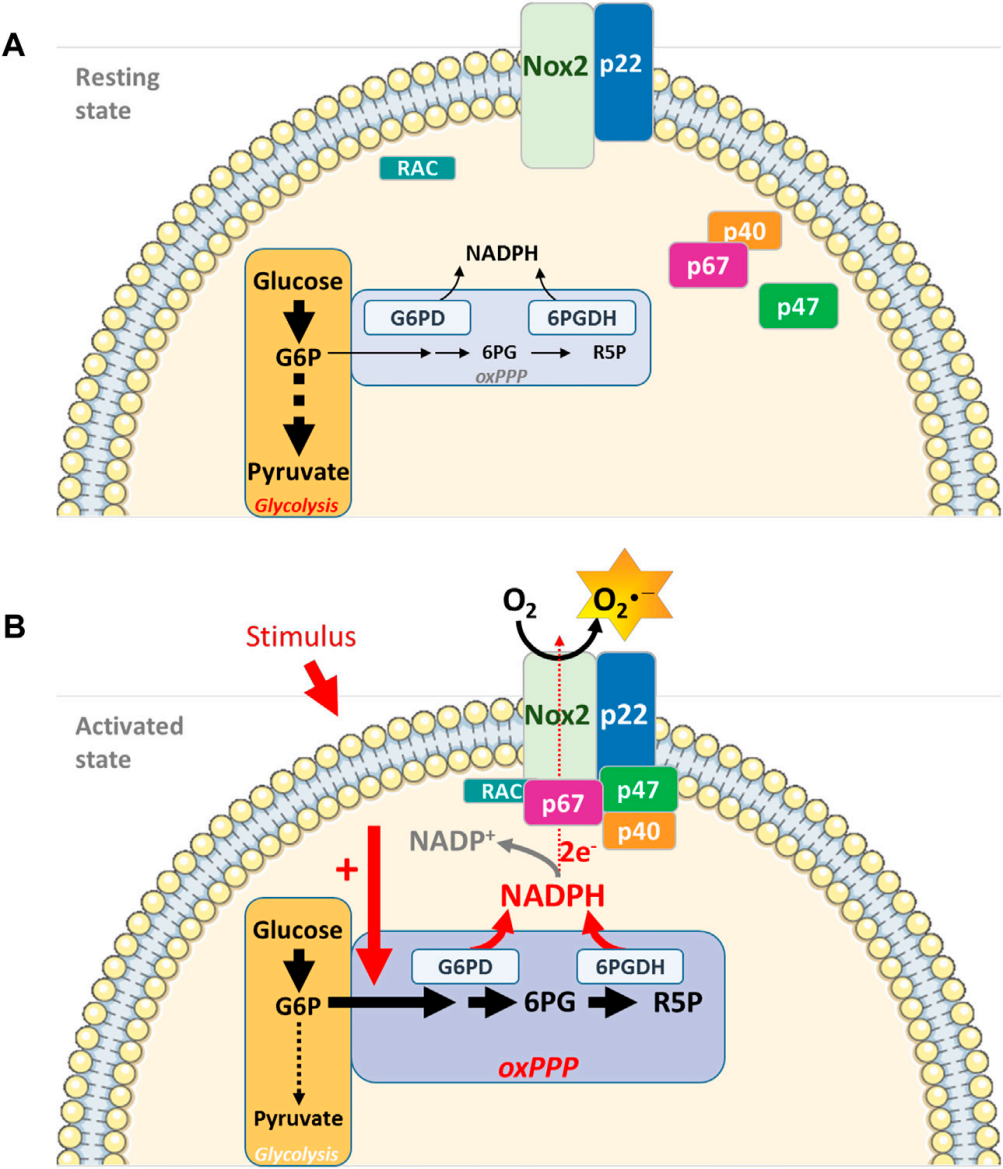
Interconnecting cell metabolism and NOX2 activity in neutrophils. **(A)**. In resting neutrophils, energy metabolism depends essentially on glucose and on the glycolytic pathway for ATP production and energy supply. NOX2 is dissociated and inactive. **(B)**. Upon cell stimulation (PMA, opsonized bacteria, fMLP), the oxidative phase of the PPP is activated, leading to an increase in NADPH concentration, a limiting cofactor for NOX2 complex activity. Micro-compartmentation coupling enzymes involved in the energy metabolism (G6PD and 6 PGDH) and NOX2 complex represent an additional level of NADPH oxidase activity regulation (G6P: Glucose-6-Phosphate; 6 PG: 6-PhosphoGluconate; R5P: Ribulose-5-Phosphate)*Parts of this figure were drawn by using pictures from Servier Medical Art. Servier Medical Art by Servier is licensed under a Creative Commons Attribution 3.0 Unported License (* *https://creativecommons.org/licenses/by/3.0/* *).*

Cellular micro-compartmentation, coupling energy metabolism to ROS production, provides an additional level of NADPH oxidase activity regulation (Figure 1).

### Phosphoinositide Dynamics and NADPH Oxidase Regulation

Metabolism contributes to the regulation of NOX2 activity whose assembly is dependent on the subunit interaction. However, not only protein-protein binding is important for NADPH oxidase activity, but also protein-lipid binding. P47^phox^ and p40^phox^ have a PX domain. The PX domain of p47^phox^ has two binding pockets: one prefers phosphatidylinositol 3,4-bisphosphate (PI(3,4)P_2_) and the other binds phosphatidic acid and phosphatidylserine, whereas the PX domain of p40^phox^ binds PI3P (Ellson et al., 2001; Kanai et al., 2001; Karathanassis et al., 2002; Stahelin et al., 2003). The phosphoinositide composition of the inner leaflet of the membrane during neutrophil activation is crucial to sustain NADPH oxidase activity. The understanding of the importance of the p40^phox^-PX for ROS production came from the discovery of a CGD patient with a mutation of a critical residue for PI3P binding in the PX domain (Matute et al., 2009). Neutrophils of this patient presented a substantial defect in intracellular ROS production during phagocytosis of *Aspergillus fumigatus* hyphae or serum opsonized fungus particles but not upon activation of neutrophils by soluble stimuli (Matute et al., 2009; Bagaitkar et al., 2012). Neutrophils of mice bearing a PX mutation in p40^phox^ also had a reduced ROS production upon phagocytosis although this depends on the stimulus (Ellson et al., 2006; Anderson et al., 2010). In p40^phox^, the PX domain is masked by an intramolecular interaction (Honbou et al., 2007) which is removed in the presence of H_2_O_2_ and when p40^phox^ is targeted to the membrane (Ueyama et al., 2011). Thus, it may be only when the p40^phox^/p67^phox^/p47^phox^ complex is at the membrane that p40^phox^ can bind to PI3P.

**FIGURE 2:**
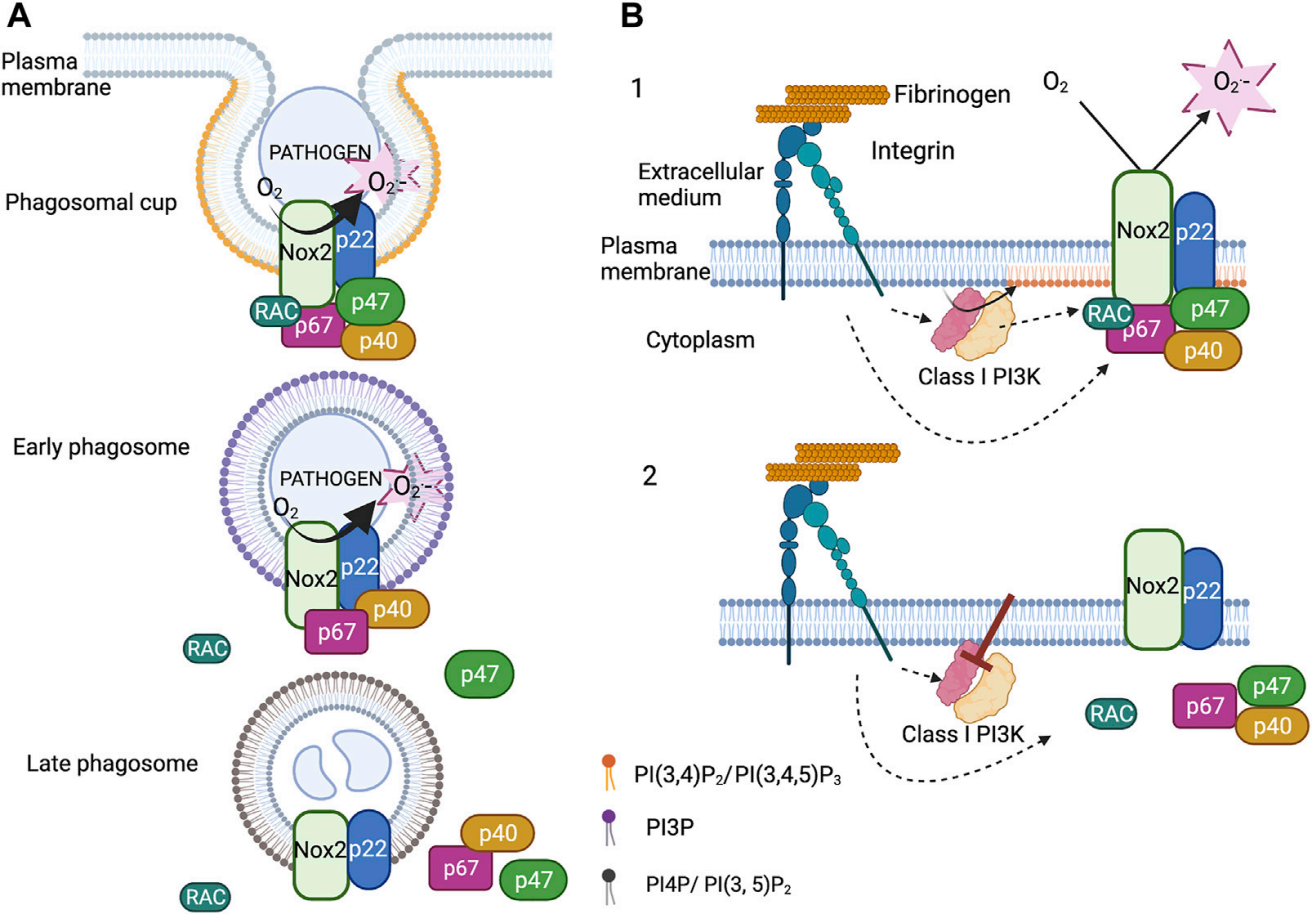
Regulation of the NADPH oxidase by phosphoinositides. **(A)**. Upon phagocytosis of serum opsonized fungus particles, PI(3,4)P_2_ and PIP_3_ accumulate in the inner leaflet of the phagosomal membrane. At the same time, the cytosolic subunits (p67^phox^, p47^phox^ and p40^phox^) and the small GTPases Rac2 associate with the membrane subunits (Nox2 and p22^phox^). Soon after phagosome closure, p47^phox^ and Rac2 leave the phagosome. The detachment of p47^phox^ is concomitant to the decrease in the level of PI(3,4)P_2_ and the accumulation of PI3P in the cytosolic leaflet of the early phagosome. The binding of p40^phox^ to PI3P sustains the NADPH oxidase activity. The disappearance of PI3P induces the disassembly of the complex. **(B)**. Integrin dependent adherent neutrophils, stimulated or not by fMLP, produce ROS *via* NADPH oxidase activation. Class I PI3Ks are also activated and are necessary to sustain NADPH oxidase activation. Inhibition of Class I PI3Ks, especially the *β* isoform, deactivate the NADPH oxidase by triggering its disassembly. Class I PI3K products maintain the cytosolic subunits at the plasma membrane probably via the PX domain of p47^phox^ (*created with “BioRender.com”*).

The NADPH oxidase complex assembles at the phagosomal cup. At this time PI (3,4)P_2_ and PIP_3_ accumulate in the inner leaflet of the phagosomal cup. This accumulation is transient and followed by the rise of PI3P in the inner leaflet 1 min after the phagosome sealing. PI3P can be generated through dephosphorylation of PI(3,4)P_2_ and by phosphorylation of phosphatidylinositol by the class III phosphoinositide 3-kinase (PI3K) (Vieira et al., 2001; Valenta et al., 2020; Montaño-Rendón et al., 2021). Using the PX of p40^phox^ tagged with GFP as a PI3P biosensor, we observed that PI3P lasted around 15min at the phagosome of opsonized fungus particles in neutrophil-like PLB-985 cells. Expressing fluorescent protein fusion of p40^phox^ and p67^phox^ in these cells allowed us to prove that the timing of their disappearance at the level of the phagosome correlated with that of PI3P. Moreover, we showed that the protein Rubicon, a negative regulator of Class III PI3K (Sun et al., 2011), and the PI3P phosphatase Myotubular Myopathy 1 (MTM1) (Cao et al., 2008; Amoasii et al., 2013), were present at the phagosome. Knocking down these two proteins increased the time that the PI3P biosensor, p40^phox^ and p67^phox^ remained present at the phagosomal membrane and also ROS production inside the phagosome. In contrast, overexpression of MTM1 at the phagosome prevented the accumulation of PI3P, p40^phox^ and p67^phox^ and ROS production. Thus, the disappearance of PI3P from the phagosomal membrane controls the disassembly of the NADPH oxidase complex and thus the ROS production inside the phagosome (Song et al., 2017) (Figure 2A).

The NADPH oxidase also assembles at the plasma membrane and produces ROS in the extracellular medium. This ROS production is involved in neutrophil migration and may have other physiological consequences. However, this can also be detrimental contributing to thrombus formation (Gutmann et al., 2020). A long-lasting NADPH oxidase activation at the plasma membrane has been observed, *in vitro*, in β2 integrin dependent adherent neutrophils stimulated or not by fMLP or TNF (Fumagalli et al., 2013; Houslay et al., 2016; Song et al., 2020b). Integrins, in their active conformation, stimulated different intracellular pathways including the production of PI(3,4)P_2_ and PIP_3_ by class I PI3K (Mócsai et al., 2015). Class I PI3K works as a heterodimer possessing p110 catalytic subunits and regulatory subunits. Two subclasses, IA and IB, can be distinguished. Subclass IA comprises three types of catalytic subunits (p110α, p110β, p110δ) that share several regulatory subunits: p85, p50 and p55 (Balla, 2013).

The pharmacological inhibition of class I PI3K in adherent neutrophils, and especially p110β, halted integrin mediated ROS production. Expression of tagged p47^phox^, p40^phox^ and p67^phox^ in neutrophil-like PLB-985 cells allowed us to observe, by total internal reflexion fluorescence video-microscopy and after class I PI3K inhibition, the release of these subunits from the plasma membrane. Our results suggest that this mechanism involves the PX domain of p47^phox^, which binds PI (3,4)P_2_ (Figure 2B). This is coherent with the fact that mutation in some critical residues involved in PI(3,4)P_2_ binding only slightly modified ROS production at the phagosome but drastically affected that at the plasma membrane following fMLP activation (Li et al., 2010; Olsson et al., 2017). Thus, Class I PI3K products may be important for the deactivation of the NADPH oxidase at the plasma membrane whereas PI3P would act on the timing of NADPH oxidase activation at the phagosome (Figure 2).

## Concluding Remarks

A fine-tuned regulation of the NADPH oxidase is necessary. Polymorphism in the NADPH oxidase genes leading to low ROS production has recently been associated with autoimmune diseases such as systemic lupus erythematosus (Olsson et al., 2017). In contrast, excess activation of neutrophils and NADPH oxidase dependent ROS production contribute to several chronic inflammatory diseases. Inhibitors of class I PI3K isoforms are currently in clinical testing or approved for drug use (Miller et al., 2019) and thus may be interesting targets in order to reduce the duration of NADPH activity at the plasma membrane. Modulating cellular metabolism by regulating the PPP pathway could be a way to moderate or, on the contrary, to increase NOX2-dependent ROS production. Furthermore, it may be of great interest to selectively target NOX2 at the plasma membrane versus phagosomal membrane.
